# Morphodynamic Features of Contrast-Enhanced Mammography and Their Correlation with Breast Cancer Histopathology

**DOI:** 10.3390/jimaging11030080

**Published:** 2025-03-13

**Authors:** Claudio Ventura, Marco Fogante, Elisabetta Marconi, Barbara Franca Simonetti, Silvia Borgoforte Gradassi, Nicola Carboni, Enrico Lenti, Giulio Argalia

**Affiliations:** 1 SOD Radiologia Materno Infantile, Senologica, Cardiologica ed Ecografica Ambulatoriale, Azienda Ospedaliero Universitaria delle Marche, Via Conca 71, 60126 Ancona, Italy; claudioventura20@gmail.com (C.V.); elisabetta.marconi@ospedaliriuniti.marche.it (E.M.); barbarafranca.simonetti@ospedaliriuniti.marche.it (B.F.S.); silvia.borgofortegradassi@ospedaliriuniti.marche.it (S.B.G.); nicola.carboni@ospedaliriuniti.marche.it (N.C.); giulio.argalia@ospedaliriuniti.marche.it (G.A.); 2 SOD Chirurgia Senologica, Azienda Ospedaliero Universitaria delle Marche, Via Conca 71, 60126 Ancona, Italy; enrico.lenti@ospedaliriuniti.marche.it

**Keywords:** contrast-enhanced mammography, CEM, breast cancer, histopathological correlation, imaging biomarkers, tumor characterization

## Abstract

Contrast-enhanced mammography (CEM) combines morphological and functional imaging, enhancing breast cancer (BC) diagnosis. This study investigates the relationship between CEM morphodynamic features and histopathological characteristics of BC. In this prospective study, 50 female patients (mean age: 57.2 ± 13.7 years) with BI-RADS 4–5 lesions underwent CEM followed by surgical excision between December 2022 and May 2024. Low-energy and recombined CEM images were analyzed for breast composition, lesion characteristics, and enhancement patterns, while histopathological evaluation included tumor size, histotype, grade, lymphovascular invasion, and immunophenotype. Spearman rank correlation and multivariable regression analysis were used to evaluate the relationship between CEM findings and histopathological characteristics. Tumor size on CEM strongly correlated with histopathological tumor size (ρ = 0.788, *p* < 0.001) and was associated with high-grade lesions (*p* = 0.017). Non-circumscribed margins were linked to a Luminal-B subtype (*p* = 0.001), while high lesion conspicuity was associated with Luminal-B and triple-negative BC (*p* = 0.001) and correlated with larger tumors (ρ = 0.517, *p* < 0.001). Background parenchymal enhancement was negatively correlated with age (ρ = −0.286, *p* = 0.049). CEM provides critical insights into BC, demonstrating significant relationship between imaging features and histopathological characteristics. These findings highlight CEM’s potential as a reliable tool for tumor size estimation, subtype characterization, and prognostic assessment, suggesting its role as an alternative to MRI, particularly for patients with contraindications.

## 1. Introduction

Breast cancer (BC) is the most common malignancy among women globally, underscoring the importance of continuous advancements in diagnostic imaging to enhance early detection, precise characterization, and effective treatment planning [1,2,3].

Among these innovations, contrast-enhanced mammography (CEM) has emerged as a promising modality, leveraging dual-energy technology to provide both anatomical and functional imaging through the use of contrast medium [4,5]. By capturing vascularization and neoangiogenesis characteristics of malignant lesions, CEM offers actionable insights that complement conventional mammography and ultrasound findings. CEM demonstrates notable advantages over magnetic resonance imaging (MRI), including shorter examination times, lower costs, and higher patient acceptability, particularly for individuals who are claustrophobic or have contraindications to MRI [6]. Studies have demonstrated that CEM achieves comparable diagnostic performance to MRI, especially in preoperative staging, screening high-risk patients with dense breasts, and assessing responses to neoadjuvant therapy [7,8,9,10]. Moreover, despite its slightly lower sensitivity compared to MRI, CEM demonstrates higher specificity, supporting its use in clinical scenarios requiring precise lesion characterization [11,12].

The standardization of CEM reporting advanced significantly with the introduction of the Breast Imaging Reporting and Data System (BI-RADS) for CEM by the American College of Radiology in 2022. The introduction of standardized descriptors has facilitated consistent reporting of findings, enabling detailed evaluation of lesion morphology and contrast enhancement patterns [13,14]. Emerging research highlights the correlation between these imaging features and histopathological characteristics, offering potential prognostic insights [15,16,17,18].

While numerous studies have explored the association between CEM findings and BC characteristics, many have not comprehensively analyzed the full spectrum of imaging features [19]. This study aims to bridge this gap by evaluating the relationship between CEM morphodynamic features and histopathological parameters, contributing to the growing body of evidence supporting CEM’s diagnostic and prognostic roles in BC management.

## 2. Materials and Methods

### 2.1. Study Design and Population

This prospective, monocentric study was approved by the Institutional Review Board (protocol identification number and approval date: 2771—15 December 2022), and written informed consent was obtained from all participants.

**Figure 1:**
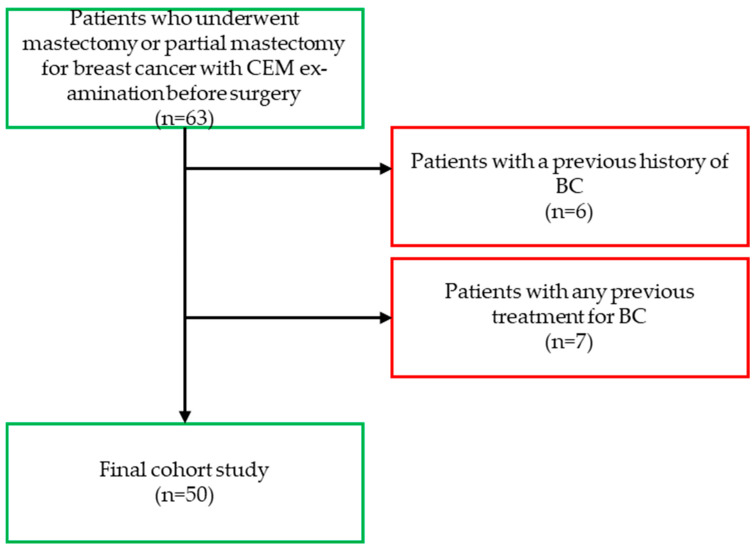
Flow-chart. Abbreviations—CEM: contrast-enhanced mammography; BI-RADS: Breast Imaging Reporting and Data System; BC: breast cancer.

Between December 2022 and May 2024, we consecutively enrolled patients who underwent mastectomy or partial mastectomy for BC with CEM examination before surgery (n = 63). The exclusion criteria were patients with a previous history of BC (N = 6) and patients with any previous treatment for BC (N = 7). For each patient, clinical, radiological, and histopathological data were collected. A total of 50 patients were included in this study (Figure 1).

### 2.2. Image Acquisition

All CEM examinations were performed using a dual-energy mammograph (Giotto Class Series 30000, Hologic, Massachusetts, USA). Following intravenous administration of a non-ionic contrast agent (iopamidol, 1.2 mL/kg at a rate of 3 mL/s) and saline solution (20 mL at a rate of 3 mL/s), paired low-energy (LE) (23–32 kVp) and high-energy (45–49 kVp) images were obtained in craniocaudal and mediolateral oblique views. Recombined images were then generated from the LE and high-energy images to highlight areas with contrast enhancement. CEM images were acquired 2 min after contrast injection and completed within 10 min.

### 2.3. Image Preprocessing

Prior to analysis, all CEM images underwent standardized preprocessing to ensure consistency in size, orientation, and calibration. LE and recombined images were adjusted for brightness and contrast using vendor-specific software to optimize lesion visibility. Spatial calibration was performed using embedded scale markers to ensure accurate tumor size measurements. Regions of interest were manually delineated by radiologists to exclude artifacts and adjacent anatomical structures, ensuring focused evaluation of lesion morphology and enhancement patterns.

### 2.4. Reader Study

Two breast radiologists with 15 years of experience independently evaluated all CEM imaging features using the American College of Radiology BI-RADS lexicon for CEM (published in 2022). Both radiologists were blinded to clinical and histopathological information. In cases of disagreement, a consensus was reached. For LE images, the following CEM imaging characteristics were analyzed in this study: breast composition (A—almost entirely fatty, B—scattered areas of fibroglandular, C—heterogeneously dense, D—extremely dense); presence of microcalcifications; and presence of architectural distortion. For recombined images, the following parameters were evaluated: size; location; shape (oval, round, irregular); margins (circumscribed, non-circumscribed); internal enhancement characteristics (homogeneous, heterogeneous, rim enhancement); lesion conspicuity (low, moderate, high); and associated features (nipple retraction, nipple invasion, skin retraction, skin thickening, skin invasion, axillary adenopathy). Moreover, background parenchymal enhancement (BPE) was evaluated. In cases with multicentric or multifocal lesions, the lesion with the largest dimension was selected for analysis.

### 2.5. Histopathological Evaluation

Pathological specimens were obtained through surgical excision and analyzed by a single pathologist with 15 years of experience in breast pathology. The following parameters were evaluated: tumor size; histotype (ductal or lobular); grade (G1, G2, or G3) determined using the Nottingham modification of the Bloom and Richardson method (Elston–Ellis criteria); presence of lymphovascular invasion; and immunophenotype (IF) classification, based on the expression of estrogen receptor (ER), progesterone receptor (PR), and human epidermal growth factor receptor 2 (HER2). Immunohistochemical (IHC) testing was used to determine receptor status. According to IHC, nuclear staining ≥10% was regarded as positive for ER and PR status. Expression of HER-2 protein was classified as 0, 1+, 2+, and 3+ by IHC. If a specimen was categorized as 2+, fluorescence in situ hybridization (FISH) was performed to test the HER-2 gene. HER-2 positivity was defined as an IHC HER-2 score of 3+ or gene amplification by FISH. HER-2 negativity was defined as an IHC HER-2 score of 0 or 1+. Based on ER, PR, HER2, and Ki-67 expressions, BC were classified into four IFs: Luminal-A (ER-positive and/or PR-positive, HER2-negative, and Ki-67 < 20%); Luminal-B (ER-positive and/or PR-positive, HER2-negative, and Ki-67 ≥ 20% or ER and/or PR-positive and HER2-positive, irrespective of Ki-67 expression); HER2-enriched (ER-negative, PR-negative, and HER2-positive); and triple-negative (ER-negative, PR- negative, and HER2-negative).

### 2.6. Statistical Analysis

All statistical analyses were performed by using the Statistical Package for the Social Sciences 21.0 (International Business Machine Corporation, Chicago, IL, USA). Continuous variables were expressed as mean ± standard deviation, while categorical variables were expressed as frequencies and percentages. Variables found to be statistically significant in the univariate analysis were further examined using a multivariable regression model to assess the relationship between CEM findings and histopathological parameters. Moreover, relationships between intra-CEM and intra-histopathology findings were evaluated. Due to the non-normal distribution of variables, Spearman rank correlation analysis was applied to identify correlations among statistically significant parameters. A *p*-value < 0.05 was considered statistically significant.

## 3. Results

### 3.1. Study Population

A total of 50 patients were included and 50 BCs were evaluated. The mean age of patients was 57.2 ± 13.7 years and 13 (26%) patients presented palpable nodules. Table 1 summarizes the main demographic and clinical characteristics of the study population.

### 3.2. CEM Findings

**Figure 2:**
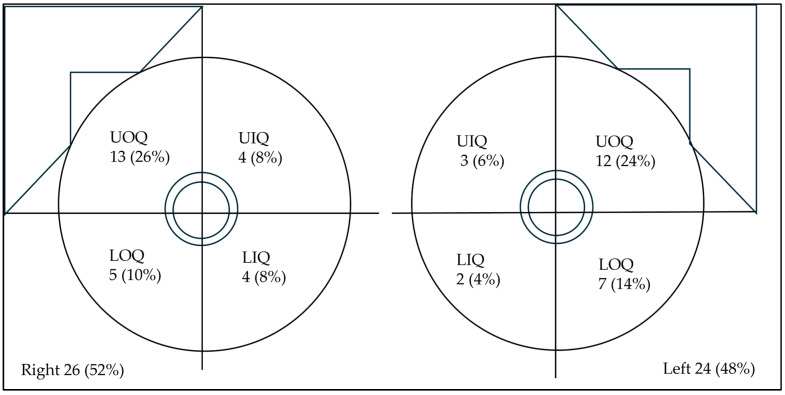
Distribution of lesions across breast quadrants. Abbreviations—UOQ: upper outer quadrant; UIQ: upper inner quadrant; LOQ: lower outer quadrant; LIQ: lower inner quadrant.

LE CEM images revealed that 60% of patients had a breast composition pattern classified as category B, while microcalcifications were observed in 11 lesions (22%). Architectural distortion was detected in 34 cases (68%). Recombined CEM images revealed an average larger cancer dimension of 23.4 ± 10.9 mm. Most lesions were located in the upper outer quadrant (50%) and demonstrated an oval shape (62%), non-circumscribed margins (86%), and heterogeneous internal enhancement (74%). High conspicuity was observed in 30% of lesions. Finally, 52% of cases showed minimal BPE. Detailed findings from LE and recombined CEM images are presented in Table 2 and Table 3. Figure 2 illustrates the distribution of lesions across breast quadrants (upper outer, upper inner, lower outer, lower inner).

### 3.3. Histopathological Findings

Histopathological analysis identified ductal carcinoma in 36 cases (72%), with grade 2 being the most frequent (50%). The predominant IF was Luminal-B (52%) and lymphovascular invasion was observed in 16 cases (32%). Pathological characteristics of the lesions are summarized in Table 4.

### 3.4. Relationship Between CEM Findings and Histopathological Characteristics

**Figure 3:**
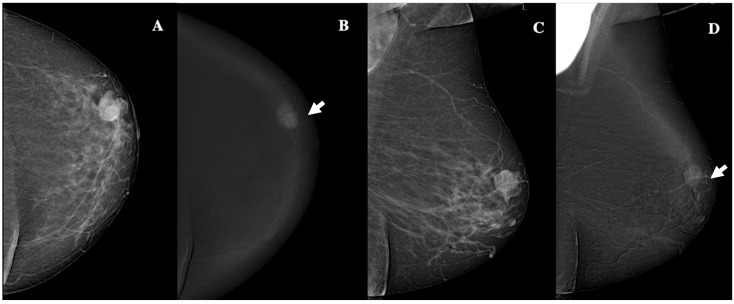
Contrast-enhanced mammography in a 60-year-old patient with BI-RADS 4 lesion. CEM revealed a 19 mm oval lesion with circumscribed margins, heterogeneous enhancement, and low conspicuity. Panel (**A**) shows the craniocaudal low-energy image, while Panel (**B**) displays the corresponding recombined image (white arrow). Panel (**C**) depicts the mediolateral oblique low-energy image and Panel (**D**) shows the recombined mediolateral oblique (MLO) image (white arrow). Histopathology identified the lesion as an invasive, grade 1, Luminal-A ductal cancer, without lymphovascular invasion.

**Figure 4:**
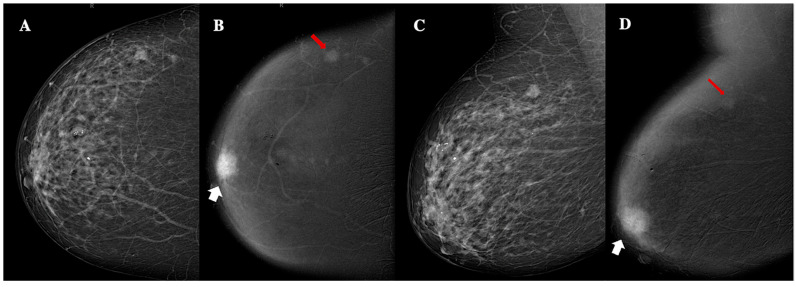
Contrast-enhanced mammography of a 63-year-old patient with BI-RADS 5 lesion. Panel (**A**,**C**) show a low-energy MLO view, highlighting dense breast parenchyma. Panel (**B**,**D**) display the corresponding recombined image, revealing a 24 mm oval lesion with non-circumscribed margins, heterogeneous enhancement, high conspicuity (white arrows), and an additional 8 mm oval lesion with circumscribed margins, homogeneous enhancement, and low conspicuity (red arrows). These findings are consistent with unilateral, multifocal, grade 3, triple-negative ductal cancer, with lymphovascular invasion.

**Figure 5:**
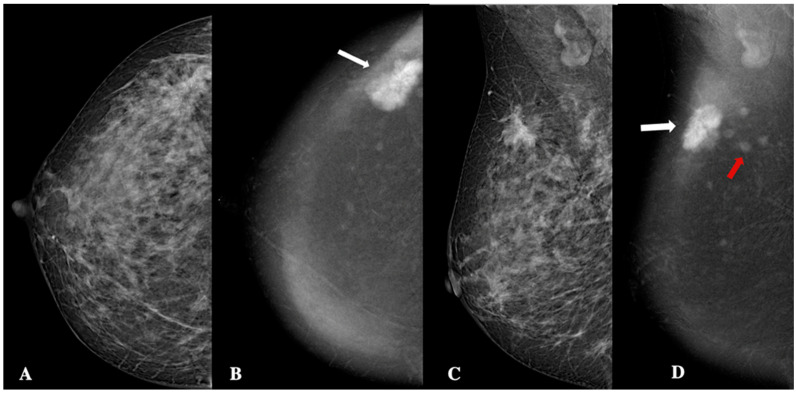
Contrast-enhanced mammography in a 42-year-old patient with BI-RADS 5 lesion. In the low-energy CEM images (panels **A**,**C**), a multilobulated area of architectural distortion with non-circumscribed margins is visible in the upper outer quadrant. This finding is more clearly delineated in the recombined CEM images (panels **B**,**D**). The main lesion, measuring approximately 24 mm, demonstrates high conspicuity and heterogeneous enhancement (white arrows). Additionally, four smaller foci, each approximately 4 mm in size, exhibit low conspicuity enhancement and sharper margins, and are considered satellite lesions (red arrow). The lesion was identified as invasive, unilateral, multifocal, grade 2, Luminal-B ductal cancer, without lymphovascular invasion.

**Figure 6:**
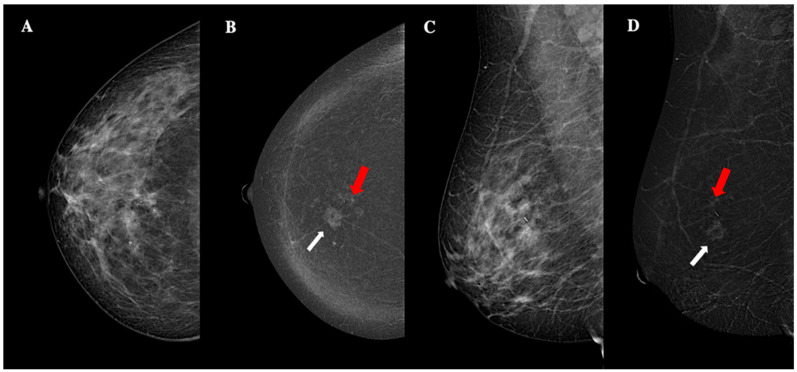
Contrast-enhanced mammography in a 54-year-old patient with BI-RADS 5 lesion. CEM low-energy images (panels **A**,**C**) show pseudonodular opacities with associated microcalcifications and ill-defined margins are observed in the lower inner quadrant. These findings are more distinctly visualized in the recombined CEM images (panels **B**,**D**). The white arrows highlight the main lesion, approximately 14 mm in size, exhibiting moderate conspicuity, heterogeneous enhancement, and ill-defined margins. Additionally, two similar foci, measuring approximately 8 mm and 4 mm, with comparable morphological features, were identified (red arrows). The lesion was diagnosed as a unilateral, multifocal, grade 3, invasive, Her2-enriched ductal cancer, without lymphovascular invasion.

**Figure 7:**
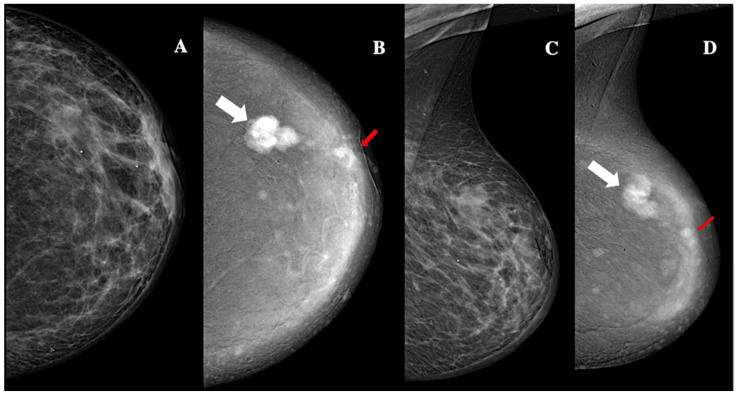
Contrast-enhanced mammography in a 76-year-old patient with BI-RADS 5 lesion. CEM low-energy images (panels **A**,**C**) showing pseudonodular opacities with signs of skin retraction are visible in the upper outer quadrant and are more clearly delineated in the recombined images (panels **B**,**D**). The main lesion, measuring approximately 21 mm, exhibits high conspicuity, heterogeneous internal enhancement, and lobulated margins (white arrows). A corresponding area of skin retraction (red arrows), approximately 9 mm in size, also demonstrates high enhancement. The lesion was diagnosed as a unilateral, multifocal, grade 3, triple-negative, invasive lobular carcinoma, with lymphovascular invasion.

No significant relationships were observed between breast composition, microcalcifications and architectural distortion, and histopathological characteristics. However, the larger tumor dimension measured on CEM was strongly correlated with larger histopathological tumor size (ρ = 0.788, 95% confidence interval (CI): 0.653–0.875, *p* < 0.001) and was associated with grade 3 tumors (*p* = 0.017). No significant associations were identified between BC side, location, shape, lesion internal enhancement, and other associated features and histopathological characteristics. Non-circumscribed margins were significantly associated with the Luminal-B (*p* = 0.001) subtype. High lesion conspicuity was strongly associated with the Luminal-B (*p* = 0.001) and triple-negative IFs (*p* = 0.001); additionally, it was positively correlated with larger histopathological tumor size (ρ = 0.517, 95% CI: 0.279–0.695, *p* < 0.001). Furthermore, BPE demonstrated a significant negative correlation with patient age (ρ = −0.286, 95% CI: −0.523, −0.008, *p* = 0.049). Table 5 summarizes the statistically significant relationships between CEM findings and histopathological/clinical characteristics. Moreover, within CEM findings, non-circumscribed margins were significantly associated with larger tumor size (*p* = 0.003). Similarly, within histopathological analysis, Luminal-B tumors correlated with a higher histological grade (*p* = 0.012). Figure 3, Figure 4, Figure 5, Figure 6 and Figure 7 show examples of patients included in our study.

## 4. Discussion

BC is the most prevalent malignancy among women worldwide, making it a critical focus for continuous innovation in diagnostic imaging. The advancements in imaging techniques have significantly impacted the early detection, characterization, and management of BC [1,20,21]. Among these innovations, CEM has gained considerable attention due to its ability to provide both morphological and functional information using contrast agents. CEM offers high specificity, shorter exam times, and better patient acceptability compared to MRI, especially for those with contraindications [5,8,9,10]. While emerging research suggests correlations between CEM imaging features and histopathological characteristics, comprehensive evaluations of CEM’s capabilities remain limited [19]. This study aims to analyze the morphodynamic features of CEM and their association with BC histopathology, thereby advancing its diagnostic and prognostic value.

CEM provided detailed imaging features that helped in identifying lesions with varying characteristics. The majority of lesions (74%) displayed heterogeneous internal enhancement, which is consistent with previous studies showing that this pattern is more commonly associated with malignancies [22]. Additionally, most lesions (86%) had non-circumscribed margins, which further supports the notion that irregular margins are indicative of invasive carcinoma. The conspicuity of lesions varied, with 50% showing low conspicuity, and 30% exhibiting high conspicuity. These findings suggest that while CEM can be helpful in visualizing malignancies, the conspicuity of the enhancement may limit its diagnostic power in some cases, similar to what has been reported in the prior literature [23].

One of the key findings of this study is that CEM imaging provides detailed information that correlates with histopathological tumor size, a crucial factor in BC staging and treatment planning. The positive correlation between larger BC size on CEM images and histopathological evaluation (ρ = 0.788, *p* < 0.001) supports the use of CEM for accurate tumor size estimation. The existing literature extensively reports on the effectiveness of CEM in detecting and characterizing BC, especially in dense breast tissue, where conventional mammography may have reduced sensitivity. This highlights CEM’s reliability in accurately sizing lesions, regardless of breast density. Accurate preoperative tumor size estimation remains critical for surgical planning and treatment decisions [24,25]. Furthermore, research has shown that CEM is not inferior to MRI in preoperative evaluations, particularly in dense breasts [10,26,27,28]. However, it is important to note that false negatives in CEM are typically observed in smaller, monofocal lesions (less than 10 mm in size) or in cases of ductal carcinoma in situ, where the only indication might be suspicious microcalcifications [15].

BPE was another feature examined in this study. We found a significant negative correlation between BPE and patient age (ρ = −0.286, *p* = 0.049), which is consistent with findings from previous studies suggesting that younger women tend to exhibit higher levels of BPE in a CEM context [15]. BPE is influenced by various factors, including hormonal status, and can impact the sensitivity of mammography and other imaging modalities.

Moreover, high lesion conspicuity on CEM was also strongly associated with both Luminal-B and triple-negative IFs (*p* = 0.001). These IFs are known for their poor prognosis and more aggressive clinical behavior. This finding corroborates previous studies highlighting the role of CEM in identifying tumors with active blood vessel formation, a hallmark of malignancy. This is probably because Luminal-B and triple-negative subtypes tend to have higher vascularity, neoangiogenesis, and increased permeability, leading to higher contrast uptake and conspicuity [23]. Moreover, the correlation between high lesion conspicuity and larger tumor size observed in our study further supports the use of CEM in assessing the extent of tumor spread, which is crucial for preoperative staging and treatment planning.

Furthermore, one of the key observations in our study was the correlation between non-circumscribed margins on CEM and Luminal-B. This is in line with the existing literature that suggests that non-circumscribed margins are indicative of more aggressive tumors and may be a manifestation of tumor cells invading surrounding tissues [23]. The significant association of non-circumscribed margins with the Luminal-B subtype further underscores the value of CEM in characterizing biologically aggressive tumors. This is especially important for guiding clinical decision-making, as Luminal-B tumors are typically more aggressive than Luminal-A tumors and may require more intensive treatment regimens.

The results from this study emphasize the potential role of CEM as a complementary imaging tool in BC diagnosis and management. In addition to histopathological correlations, this study highlights the advantages of CEM in terms of diagnostic efficiency and patient acceptability. CEM offers a significant advantage over traditional mammography in patients with dense breasts, where conventional mammography may fail to identify lesions effectively. It also provides a viable alternative for patients who are unable to undergo MRI due to contraindications such as claustrophobia. The ability to capture both anatomical and functional imaging information within a single exam, with relatively short examination times, makes CEM an attractive option for clinical practice. Furthermore, the use of a standardized reporting system such as BI-RADS for CEM enhances its consistency and reproducibility, ensuring that radiologists can reliably communicate findings across clinical settings. However, it is necessary to consider the limits of CEM, specifically with regard to its sensitivity in the detection of small lesions and DCIS that may have subtle or absent enhancement on CEM. Moreover, MRI remains superior in evaluating non-mass enhancements and identifying lesions with low vascularity, which may not exhibit strong contrast uptake in CEM [12].

Finally, emerging studies suggest artificial intelligence (AI) integration into CEM analysis. Advances in AI and machine learning could significantly enhance CEM’s diagnostic accuracy and prognostic value by analyzing complex patterns within imaging data that are not immediately apparent to human observers [29,30,31,32].

The database collected in this study lays the groundwork for several promising research directions, including multicenter validation, comparative studies, and longitudinal prognostic analyses. Future investigations could explore associations between CEM features and long-term outcomes, such as recurrence-free survival or treatment response.

Despite the promising results, this study has some limitations. The sample size is relatively small (n = 50), which may limit the generalizability of the findings, particularly regarding histological subtypes. While our study provides valuable preliminary insights, we recognize the need for larger, multicentric studies to validate these correlations across diverse patient populations. Additionally, the study was monocentric, and the findings might vary in a multicenter setting with a more diverse population. Moreover, this study did not assess interobserver variability among radiologists evaluating CEM images, which is an important factor in establishing the consistency and reliability of CEM-based diagnoses. The absence of smaller or non-enhancing lesions in our study sample may have introduced some selection bias. Finally, the histopathological evaluation was conducted by a single pathologist, introducing potential for observer bias or diagnostic variability. While the pathologist has extensive experience (15 years in breast pathology), the lack of interobserver validation may affect result generalizability. Future studies should incorporate multiple blinded pathologists to assess inter-rater reliability.

## 5. Conclusions

In conclusion, CEM proves to be a versatile, patient-friendly imaging technique with considerable diagnostic and prognostic capabilities. Its correlation with histopathological tumor characteristics, particularly the enhancement of biologically aggressive lesions, highlights its clinical value. This suggests that CEM can provide valuable insights into tumor characteristics, potentially aiding in diagnosis, staging, and treatment planning. With ongoing technological advancements and larger-scale studies, CEM has the potential to become a key component of BC imaging in clinical practice, complementing existing modalities and offering deeper insights into tumor biology.

## Figures and Tables

**Table 1:** Demographic and clinical characteristics of the study population.

**Demographic characteristics**	Age (year, mean ± SD)	57.2 ± 13.7
	Body mass index (kg/m^2^, mean ± SD)	23.7 ± 8.1
**Clinical characteristics**	Presence of palpable nodule (n, %)	13 (26%)
	Presence of nipple secrection (n, %)	3 (6%)
	Presence of retraction (n, %)	2 (4%)
	Presence of cutaneous retraction (n, %)	3 (6%)
	Presence of palpable adenopathy (n, %)	4 (8%)
	Family history of breast cancer (n, %)	17 (34%)

Abbreviations—SD: standard deviation.

**Table 2:** Key findings from LE CEM images.

**Breast composition**	A (almost entirely fatty) (n, %)	4 (8%)
	B (scattered fibroglandular areas) (n, %)	30 (60%)
	C (heterogeneously dense) (n, %)	14 (28%)
	D (extremely dense) (n, %)	2 (4%)
**Microcalcifications**	Present (n, %)/absent (n, %)	11 (22%)/39 (78%)
**Architectural distorsion**	Present (n, %)/absent (n, %)	34 (68%)/16 (32%)

**Table 3:** Key findings from recombined CEM images.

**Mean lesion dimension**	Size (mm—mean ± SD)	23.4 ± 10.9
**Lesion side**	Right (n,%)/left (n, %)	26 (52%)/24 (48%)
**Lesion location**	UOQ (n,%)/UIQ (n,%)	25 (50%)/7 (14%)
	LOQ (n,%)/LIQ (n,%)	12 (24%)/6 (12%)
**Lesion shape**	Oval (n, %)/round (n, %)/irregular (n,%)	31 (62%)/9 (18%)/10 (20%)
**Lesion margins**	NC (n, %)/C (n, %)	7 (14%)/43 (86%)
**Lesion internal enhancement**	Homogeneous (n, %)	8 (16%)
	Heterogeneous (n, %)	37 (74%)
	Rim enhancement (n, %)	5 (10%)
**Lesion enhancement conspicuity**	Low (n, %)	25 (50%)
	Moderate (n, %)	10 (20%)
	High (n, %)	15 (30%)
**Associated features**	Nipple retraction (n, %)	4 (8%)
	Nipple invasion (n, %)	2 (4%)
	Skin retraction (n, %)	2 (4%)
	Skin thickening (n, %)	3 (6%)
	Skin invasion (n, %)	9 (18%)
	Axillary adenopathy (n, %)	3 (6%)
**Background parenchymal enhancement**	Minimal (n, %)	26 (52%)
	Mild (n, %)	15 (30%)
	Moderate—marked (n, %)	9 (18%)

Abbreviations—SD: standard deviation; UOQ: upper outer quadrant; UIQ: upper inner quadrant; LOQ: lower outer quadrant; LIQ: lower inner quadrant; NC: non-circumscribed; C: circumscribed.

**Table 4:** Pathological characteristics of breast lesions.

**Mean lesion dimension**	Size (mm) (mean ± SD)	23.7 ± 14.3
**Histological phenotype**	Ductal (n, %)	36 (72%)
	Lobular (n, %)	14 (28%)
**Grading (Elston–Ellis)**	Grade 1 (n, %)	7 (14%)
	Grade 2 (n, %)	25 (50%)
	Grade 3 (n, %)	18 (36%)
**Immunophenotype**	Luminal-A (n, %)	18 (36%)
	Luminal-B (n, %)	26 (52%)
	HER2-enriched (n, %)	2 (4%)
	Triple-negative (n, %)	4 (8%)
**Lymphovascular invasion**	Present (n, %)/absent (n, %)	16 (32%)/34 (68%)

Abbreviations—SD: standard deviation; HER2: human epidermal growth factor receptor 2.

**Table 5:** Statistically significant relationships between CEM findings and histopathological/clinical characteristics.

CEM Findings	Histopathological/Clinical Characteristics	Statistical Relationship
**Tumor dimension**	Tumor size	Positive correlation (ρ = 0.788, *p* < 0.001)
	Grade 3	Significant association (*p* = 0.017)
**Non-circumscribed margins**	Luminal-B	Significant association (*p* = 0.001)
**High lesion conspicuity**	Luminal-B (n, %)	Significant association (*p* = 0.001)
	Triple-negative (n, %)	Significant association (*p* = 0.001)
	Tumor size	Positive correlation (ρ = 0.517, *p* < 0.001)
**BPE**	Age	Negative correlation (ρ = −0.286, *p* = 0.049)

Abbreviations—CEM: contrast-enhanced mammography; BPE: background parenchymal enhancement; ρ: Spearman rank correlation coefficient.

## Data Availability

The data presented in this study are available upon request from the corresponding author.
